# Innovative and Community-Driven Communication Practices of the South Carolina Cancer Prevention and Control Research Network

**DOI:** 10.5888/pcd11.140151

**Published:** 2014-07-24

**Authors:** Daniela B. Friedman, Heather M. Brandt, Darcy A. Freedman, Swann Arp Adams, Vicki M. Young, John R. Ureda, James Lyndon McCracken, James R. Hébert

**Affiliations:** Author Affiliations: Heather M. Brandt, Swann Arp Adams, James Lyndon McCracken, James R. Hébert, University of South Carolina, Columbia, South Carolina; Darcy A. Freedman, Case Western Reserve University and the Prevention Research Center for Healthy Neighborhoods, Cleveland, Ohio; Vicki M. Young, South Carolina Primary Health Care Association, Columbia, South Carolina; John R. Ureda, Insights Consulting, Inc, Columbia, South Carolina.

## Abstract

The South Carolina Cancer Prevention and Control Research Network (SC-CPCRN) is 1 of 10 networks funded by the Centers for Disease Control and Prevention and the National Cancer Institute (NCI) that works to reduce cancer-related health disparities. In partnership with federally qualified health centers and community stakeholders, the SC-CPCRN uses evidence-based approaches (eg, NCI Research-tested Intervention Programs) to disseminate and implement cancer prevention and control messages, programs, and interventions. We describe the innovative stakeholder- and community-driven communication efforts conducted by the SC-CPCRN to improve overall health and reduce cancer-related health disparities among high-risk and disparate populations in South Carolina. We describe how our communication efforts are aligned with 5 core values recommended for dissemination and implementation science: 1) rigor and relevance, 2) efficiency and speed, 3) collaboration, 4) improved capacity, and 5) cumulative knowledge.

## Introduction

The Cancer Prevention and Control Research Network (CPCRN) is a national network of academic, public health, and community partners working collaboratively to reduce the burden of cancer, especially among disenfranchised and medically underserved populations (1). The mission of the CPCRN is to accelerate the adoption of evidence-based strategies for cancer prevention and control in communities through increased understanding of the dissemination and implementation (D&I) process. *Dissemination* is “the targeted distribution of information and intervention materials to a specific public health or clinical practice audience” (2,3). *Implementation* uses “strategies to adopt and integrate evidence-based health interventions and change practice patterns within specific settings” (3). Public health interventions are more effective when they expand beyond specific, isolated health-related behaviors to consider the broader social, contextual, and policy changes in the environment in which the interventions are being conducted (4,5).

The main goals of the South Carolina CPCRN (SC-CPCRN) are to disseminate, implement, and evaluate public health programs and interventions to address cancer-related health disparities, and to engage community partners and stakeholders to increase the cancer prevention and control evidence base with the intention of increasing cancer screenings, physical activity, and access to and consumption of healthful foods among high-risk and disparate populations (6,7). To achieve these goals, the SC-CPCRN collaborates with the National Association of Community Health Centers, the South Carolina Primary Health Care Association (SCPHCA), and federally qualified health centers (FQHCs). Through established partnerships with our target audiences and these stakeholders, the relevance of our efforts is strengthened (6) leading to improved health outcomes (7,8). Our community-based participatory research (CBPR) approach increases the validity and relevance of our work, ultimately providing all stakeholders with valuable information about strategies and programming for improving health and reducing cancer-related health disparities (9–12). Few interventions have employed multiple communication channels and marketing strategies that have proven effective at improving knowledge and behaviors (13,14).

We present the innovative communication efforts being conducted and evaluated by the SC-CPCRN for improving overall health and reducing cancer-related health disparities among high-risk and disparate populations across the state, in particular within African-American and rural communities. We describe how our efforts are aligned with Glasgow and colleagues’ 5 core values recommended for D&I science (2):

1. **Rigor and relevance**: focusing on diverse settings and underserved populations; using alternative research designs combined with environmental and community data
2. **Efficiency and speed**: using methods that rapidly inform decision making and implementation in health care practices
3. **Collaboration**: using a science-based team approach, partnering with communities and clinical settings, using CBPR
4. **Improved capacity**: making emerging methods and trainings available to researchers, stakeholders, and community partners
5. **Cumulative knowledge**: using current and new resources with respect to D&I science

Three initiatives from our research are described: 1) visual representation of geospatial mortality-to-incidence ratio (MIR) and health outcomes data, 2) integrating a farmers market intervention within an FQHC’s clinical system, and 3) creating a documentary film about an FQHC-based farmers market. All research with human subjects was approved by the University of South Carolina’s institutional review board.

## Mapping Cancer Mortality-to-Incidence Data

This research aimed to describe the association between the density of FQHCs with the health indicator MIR for 4 major cancers: breast, cervical, prostate, and colon. National data were obtained and linked from the national repositories of the National Cancer Institute (NCI) and the Unified Data System of the National Association of Community Health Centers. Modeling analyses were conducted and geographic information systems (GIS) mapping techniques were used to graphically display the association for all cancer sites. We found that as the density of FQHCs increased in a county, the MIR significantly decreased.

### Rigor and relevance

Integrating geospatial information with clinical practice and cancer screening and health outcomes represents a paradigm shift in the way we consider cancer prevention and control and represents another way to examine racial and ethnic cancer-related health disparities. For this initiative, we provided visually appealing maps that allowed clinical partners and key stakeholders to easily identify areas of “positive” impact of the FQHCs and areas where additional intervention (in this case, increasing FQHC density) could be beneficial. The use of statistical modeling of the relationship added further credence to these visuals. Data were available that allowed for stratification by race, rurality, and areas where there are shortages of health care professionals, which enabled further elucidation of the effect among these diverse and underserved populations. The ecological design of this study limited the interpretation of these findings; however, they indicate promising directions for future study about the potential role of FQHCs in cancer prevention and control.

### Efficiency, speed, and collaboration

By using publicly available data in unique ways (ie, the MIR calculations and GIS mapping), we conducted analyses efficiently in 2 months that otherwise would have been much more cumbersome (and probably prohibitively expensive) if new surveys and participant recruitment were required. The key to the collaborative effort was the partnership with the SCPHCA. Early meetings of the research workgroup involved discussions of the data needs of FQHCs at the state and national levels. It became clear that impact evaluation results were the most important need and hence formed the basis of the goals. On the basis of our conversations with the SCPHCA and FQHC partners, we learned that FQHC staff were interested in assessing patient outcomes for FQHCs (eg, Papanicolaou tests for cervical cancer screening and laboratory prostate-specific antigen tests for prostate cancer screening) in relationship to national standards for the provision of quality primary health care. This interest became more relevant as changes occurred in the health care environment, such as the passing of the Affordable Care Act (15).

### Improved capacity and cumulative knowledge

Additional analysis of local FQHC data has been conducted on the basis of feedback and recommendations from the SCPHCA and FQHC partners. In presenting the findings from these analyses, clinical partners have used results to evaluate the cancer-specific services they provide and as the foundation for potential refinement of clinical policies and procedures. Partnering with both SCPHCA and the individual health centers also permits the SC-CPCRN to develop long-lasting, sustainable community relationships that support our team’s CBPR approach and enhances the ability of the network to implement and disseminate further evidence-based research interventions.

## Clinical Integration of a Farmers Market Intervention

The SC-CPCRN research program included the development and implementation of the first FQHC-based farmers market in the state. Two “produce prescription” programs were developed to increase patient–provider communication about the benefits of the farmers market and reduce economic barriers to using this new food access point among health center patients (14). We also developed and disseminated an implementation manual that described the process of forming health center–based farmers markets (ie, “farmacies”) (16).

### Rigor and relevance

The farmers market was strategically developed at an FQHC in an area with high rates of diet-related health conditions and low access to retailers of healthful foods, determined by using existing databases such as the Behavioral Risk Factor Surveillance System and the US Department of Agriculture Food Atlas (8,11). A CBPR approach was used that allowed stakeholder feedback to inform research efforts.

The study protocol included a prescription program that provided $1 off produce purchases made by patients redeeming their prescriptions at the farmers market. Within several weeks of the farmers market season, the participating FQHC developed their own produce prescription program that provided $5 coupons to the farmers market for patients attending diabetes management classes at the health center. Outcomes resulting from evaluations conducted at the farmers market informed future programming (eg, marketing strategies, vendor policies, additional incentive programs).

### Efficiency, speed, and collaboration

In 2011, a farmers market advisory council that included representatives from the FQHC (eg, directors of nursing, health education, facilities) was formed and still exists. This group received regular (sometimes weekly) feedback about prescription program usage, which was documented through sales receipts recorded at the farmers market. This feedback loop influenced the development of the second produce prescription program developed by the FQHC as well as an additional monetary incentive program designed for recipients of the Supplemental Nutrition Assistance Program (SNAP), a federal food assistance program that provides nutrition assistance to eligible low-income individuals and families. Data also were presented at local meetings (eg, South Carolina Farmers Market Manager Conference) and to local advocates. Ultimately, a data-informed policy was passed in South Carolina in 2013 to provide monetary incentives for SNAP recipients shopping at farmers markets (17).

This project has made emerging intervention methods and training available to researchers, stakeholders (eg, primary care clinics), and community partners. These partners include community groups interested in promoting organic farming across the state.

### Improved capacity and cumulative knowledge

In March 2013, the Right Choice, Fresh Start (RCFS) team released a manual focused on forming a health center–based farmers market. The farmers market implementation manual, *Building Farmacies: A Guide for Implementing a Farmers’ Market at a Community Health Center,* designed for community leaders, practitioners, and researchers, is free and is being publicized through various listserves. In the manual, we describe our experiences using a community-engaged approach to develop the RCFS farmers market, from initial conceptualization to sustaining a health center–based market (16). It was disseminated by targeting existing Web-based communication channels among relevant stakeholder groups (eg, public health, nutrition, health center, farming and agriculture, social work). This information was spread to other networks, which then spread to additional networks. From July through December 2013, the manual was downloaded 271 times from the hosting website (http://cosw.sc.edu/component/rsform/form/17-manual-request-form).

## Documentary Film: “Planting Healthy Roots: A Look at the Right Choice, Fresh Start Farmers Market”

Health communication strategies encompassing the use of media can have a profound influence on the success of public health initiatives (18). There has been increased use of documentary films, theater, and the arts (including storytelling and telenovelas) to inform and motivate high-risk communities and to measure changes in health behavior and health outcomes (19–21). These efforts have resulted in increased awareness and knowledge, improved communication about cancer with family and friends, intention to change behaviors, and behavior change (eg, increased screening) (18–21). 

This documentary film features the process of forming and implementing the RCFS farmers market intervention. To produce the documentary film, the coalition model of filmmaking (22,23), consistent with a CBPR approach, was used. Interactive filming, producing, and editing strategies were used to produce the final version. The result was an 18-minute documentary film that captured the complexities of CBPR specifically applied to implementing an innovative farmers market. The film served as a form of process evaluation of the implementation of the farmers market and as a tool to promote and celebrate the farmers market in the community.

### Rigor and relevance

The film was released in October 2011. First, from October 2011 to January 2012, 3 community film screenings took place: 2 in the rural community in which filming took place, including showings at a local university and church; and 1 in Columbia, the capital of South Carolina, at a film festival focused on food that was sponsored by an independent theater. Attendees completed evaluation forms before and after watching the film to assess their perspectives on the quality and content of the film and the influence of the film on igniting personal and collective action. Second, from December 2011 to March 2012, the documentary film was promoted online through professional organizations (via listserves) and personal contacts to increase interest in establishing similar farmers markets and share the experience of this specific farmers market. Initial access to the film required completing a pretest, and posttest evaluations were sent approximately 4 months later to assess use of the film. We recognize the limited value of a pretest–posttest survey assessment and recommend additional measures of behavioral or health outcomes in future work (eg, changes in hemoglobin A1c among people with diabetes or prediabetes).

### Efficiency, speed, and collaboration

The documentary film was shared through public screenings and distributed to individuals and organizations requesting the film. Following evaluation of initial requests online, the documentary film was posted to YouTube, Vimeo, and SC-CPCRN and SCPHCA websites for open access. Copies of the film on DVD also were distributed during presentations about the farmers market at professional and community meetings and provided to community partners. At the 2012 annual meeting of the American Public Health Association, the film was featured as part of its Film Festival (24).

Two student filmmakers, under the direction of 2 researchers, a senior filmmaker, and a market Community Advisory Council, used personal stories, community profiles, and expert interviews to describe the formation and implementation of the market in collaboration with an FQHC in rural South Carolina. The film includes live footage with various people involved with forming the farmers market (eg, farmers, health center staff, customers, researchers). Filmmakers and stakeholders worked together through each stage of the documentary’s evolution from conceptualization through to evaluation (22,23). This approach aligns with CBPR and allows for the narrative of the film to emerge from the voices of the people in the film, who were primarily members of the community.

### Improved capacity and cumulative knowledge

In addition to dissemination strategies in the local community, the SC-CPCRN received over 400 national and international requests for the original documentary film. Copies of the DVD were distributed to 42 states, the District of Columbia, and 6 countries. Through the iterative process of creating and distributing the film, enhanced recognition of the role of community members in implementing and sustaining the farmers market was realized. The film served as a powerful visual means of portraying the process of engaging the community and promoting the farmers market (25).

## Conclusion

Although the focus of the SC-CPCRN is cancer-related, the approaches presented (ie, visual display of MIR data, farmers market prescription initiative, instruction manual, and documentary film) have the potential to decrease the burden of other chronic conditions. Close partnerships with clinical and community partners have enhanced the scope and utility of our work. Without the guiding influence of the national and local clinical providers, the statistical MIR analyses proposed within the academic “ivory tower” would never have achieved the impact that it has. Clinical partners were able to provide context and practical applications. By using data that already exist as part of standard clinical operations, preparing reports, including visual representation of findings and evaluations, was quick and efficient.

The farmers market intervention, instruction manual, and documentary film were appropriate strategies to use in conjunction with CBPR approaches to document, evaluate, and disseminate process and results. Active engagement of the community partners from the exploratory planning phases through the evaluation and dissemination phases contributed to an enhanced sense of ownership. The documentary film, for example, was conceived as a method of process evaluation but evolved to encompass cultivation of such ownership and promotion of the farmers market. Additional grants were secured by the farmers market Community Advisory Council to sustain the market and improve economic opportunity for farmers. Using innovative communication strategies that align with D&I core values will ensure that information reaches high-risk populations and policy makers across the state and that effective interventions continue to be funded.
